# Comparative study of the stability of bimatoprost 0.03% and latanoprost 0.005%: A patient-use study

**DOI:** 10.1186/1471-2415-8-11

**Published:** 2008-06-11

**Authors:** Mauricio D Paolera, Niro Kasahara, Cristiano C Umbelino, John G Walt

**Affiliations:** 1 Private practice, São Paulo, Brazil; 2 Global Health Outcomes Strategy & Research, Allergan, Inc., Irvine, CA, USA

## Abstract

**Background:**

The stability of ophthalmic preparations in multidose containers is influenced by the preservative as well as the stability of the active ingredient. Unstable drugs may require refrigeration to preserve their active ingredient level and they are more likely to degrade over time, therefore becoming more susceptible to degradation based on patient mishandling. The purpose of this study was to determine the degree of molecular degradation that occurs in bimatoprost and latanoprost in a patient-use setting.

**Methods:**

This was an open-label, laboratory evaluation of the relative stability of bimatoprost and latanoprost. Patients presently using bimatoprost (n = 31) or latanoprost (n = 34) were identified at 2 clinical sites in Brazil. Patients were instructed to use and store their drops as usual and return all used medication bottles between day 28 and day 34 after opening.

**Results:**

Bimatoprost demonstrated no degradation, but latanoprost degraded at various levels. The mean age of bimatoprost was 43.0 ± 3.4 days and the mean age of latanoprost was 43.9 ± 2.8 days (P = .072). The mean percentage of labeled concentration was 103.7% in the bimatoprost bottles and 88.1% in the latanoprost bottles (P < 001).

**Conclusion:**

This study showed that bimatoprost maintained ≥100% concentration throughout the study period while latanoprost did not.

## Background

The stability of ophthalmic preparations in multidose containers is influenced by the preservative as well as the stability of the active ingredient [1]. Unstable drugs may require refrigeration to preserve their active ingredient level, therefore becoming more susceptible to degradation based on patient mishandling. Molecular degradation, particularly among ocular hypotensive medications, can occur soon after the container is opened resulting in the loss of intraocular pressure (IOP)-lowering efficacy and possibly increasing toxicity.

Numerous clinical studies show that the synthetic prostamide analog, bimatoprost, is a highly efficacious IOP lowering agent [2-4]. Bimatoprost is a stable molecule that does not require refrigeration. Studies show that the prostaglandin analog pro-drug, latanoprost, also effectively lowers IOP [5,6]. However, the active molecule used in commercially available formulations is unstable and requires refrigeration prior to use [7,8]. Latanoprost is sensitive to extremes in light and temperature [8], and thus may be particularly susceptible to degradation in suboptimal storage conditions, which may occur in a home setting or while a patient is traveling.

Whether temperature and humidity conditions found in actual practice environments, which vary across geographic locations and seasons, induce the degree of drug degradation found under laboratory conditions is not known. The purpose of this study was to determine whether storage and use practices of bimatoprost and latanoprost IOP-lowering medications affect the degradation rate of the active molecules of these drugs. Sao Paolo, Brazil, where the medications were used, is located in an equatorial climate zone characterized by a hot, dry winter season [9]. Similar to the hot summer in many parts of the United States and Mediterranean European regions, most homes are not air conditioned when no one is home during the day.

## Methods

This was an open-label, laboratory evaluation of the relative stability of bimatoprost and latanoprost. Patients presently using bimatoprost (n = 31) or latanoprost (n = 34) were identified at two clinical sites in Sao Paulo, Brazil. After granting informed consent, all patients were instructed to store and use their eye drops at room temperature and to return all used medication bottles between day 28 and day 34 after opening. The bottles were collected between May and July 2002.

### Quantitative Methods

Once patients returned the used bottles, they were collected and stored at room temperature for subsequent shipment to a central independent laboratory (Cardinal Health, San Diego, CA). During shipping (next-day air), the bottles were stored in insulative packaging with ice packs. The bottles arrived at the testing facility between day 35 and day 42. Shipment and arrival of bottles was scheduled so that all bottles were tested on approximately day 42 after opening.

All methods were validated by the testing facility, and analyses were carried out in accordance with good laboratory practices. High performance liquid chromatography (HPLC) was used to separate the molecules of interest from degradation byproducts and synthetic impurities, and for quantitation. The method for bimatoprost (AGN 192024) used a Waters Symmetry^® ^C18 reverse-phase column, UV detection at 210 nm, and a mobile phase of 72/18/10 (water/acetonitrile/methanol, v/v/v) containing 0.03% (w/v) trifluoroacetic acid. Recovery studies were conducted for commercially available bimatoprost 0.03% preserved ophthalmic solution. Studies performed at 80%, 100%, and 125% of the label claim yielded recoveries of 100.5%, 100.6%, and 100.2% for peak areas and 101.5%, 100.6%, and 100.4% using peak heights, respectively. Linearity for bimatoprost was demonstrated from 0.002% to 0.009% (w/v) bimatoprost after dilution (equivalent to 40% to 150% of the AGN 192024 label claim after dilution). Correlation coefficients (r) of 0.9999 by peak areas and 0.9992 by peak heights were obtained and a single point standard was used for calculations using peak areas and peak heights.

For latanoprost, the method employed a Waters 600S equipped with a Waters 626 pump, a Waters 486 detector, and a Waters 717 Plus autosampler or equivalent, UV detection at 205 nm and mobile phase of 6/1/13 (water/tetrahydrofuran/methanol, v/v/v) containing 0.05% (w/v) trifluoroacetic acid. Latanoprost stock solution with an acceptable concentration between 0.45 and 0.55 μg/mL was used in recovery studies; representing a range from 90% to 110% of the labelled concentration of samples after 100-fold dilution. A Free Acid Standard, Latanoprost Standard, Latanoprost and Free Acid Mixed LOQ Standard, and Latanoprost and Free Acid Mixed Standard were run against the diluted samples to determine the latanoprost concentration. A bracketing standard was injected after every 6-sample injection. The area of the latanoprost and free acid peaks within this bracketing standard were within 5% of the average area for the initial five injections of the latanoprost and free acid peaks. The relative standard deviation (RSD) for the 5 injections of the latanoprost and free acid mixed standard were ≤2% for each peak. Latanoprost concentration was determined using the following formula:

Latanoprost concentration (μg/mL) = (average area latanoprost in sample)*(standard latanoprost concentration, μg/mL)/(average area latanoprost in standard).

The primary endpoint, percentage of labeled concentration in the bottles at the time of testing, was calculated as: (concentration of intact drug in bottle/concentration of the drug indicated on the label) × 100. Percent concentrations then were recorded for each bottle of bimatoprost and latanoprost.

### Statistical Analysis

Between-group comparisons were made using paired t-tests. The a priori level of significance for all tests was 0.05. The percentage of label claim was calculated as concentration remaining in the bottle/label claim × 100.

## Results

The bottles were collected between May and July 2002, which corresponds to the end of fall and the beginning of the dry winter season in Sao Paulo. The average ambient temperature ranged between 14°C and 24°C.

**Figure 1:**
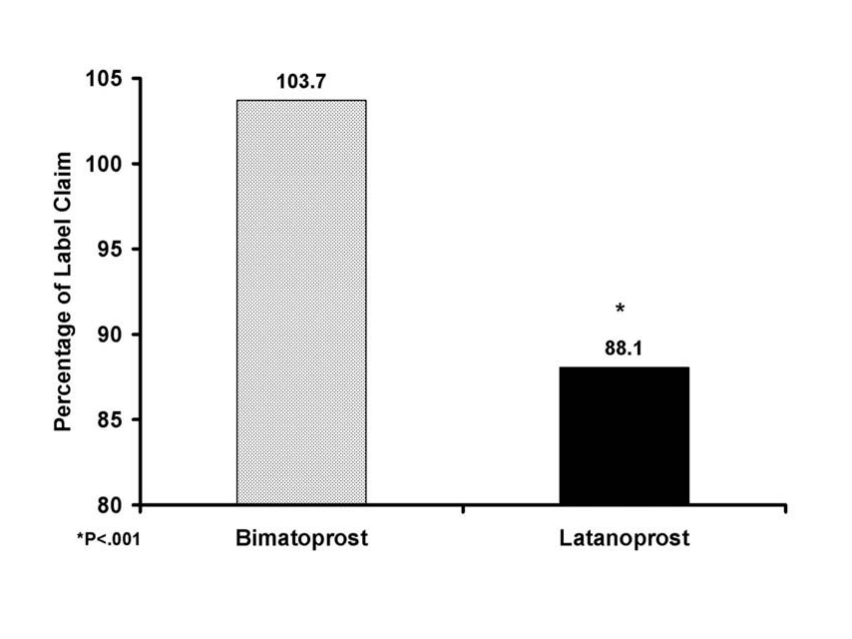
**Mean percentage of concentration in bottles relative to the label claim at time of testing**. The mean percentage (± SD) of labeled concentration was 103.7% ± 1.3% in the bimatoprost bottles compared with 88.1% ± 10.8% in the latanoprost bottles (*P *< .001)

**Figure 2:**
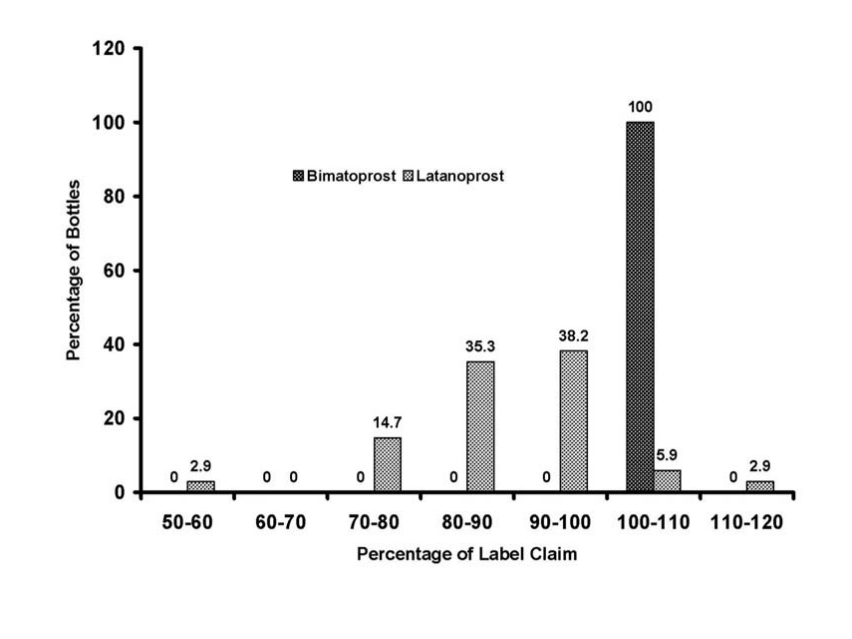
**Frequency distribution of percentage of labeled concentration**. All of the bimatoprost bottles maintained at least 100% of their labeled concentration, whereas latanoprost concentrations varied.

There was no significant between-group difference in the mean age of the bottles on the test date. The mean bottle age was 43.0 ± 3.4 days with bimatoprost and 43.9 ± 2.8 days with latanoprost (*P *= .072). Bimatoprost bottles retained a significantly greater percentage of the labeled active drug concentration after patients completed their course of therapy than did the latanoprost bottles. The mean percentage (± SD) of labeled concentration was 103.7% ± 1.3% in the bimatoprost bottles compared with 88.1% ± 10.8% in the latanoprost bottles (*P *< .001) (Figure 1), and all (31/31) of the bimatoprost bottles contained ≥100% of the labeled concentration, compared with 8.8% (3/34) of the latanoprost bottles at study end. The percentage of labeled concentration in the latanoprost bottles ranged from 52% to 115% (Figure 2). Regression analysis showed that there was no correlation between the age of the bottles at the time of testing and the percentage of labeled concentration (r^2 ^= .009, *P *= .459).

## Discussion

This study demonstrated that bimatoprost maintained its labeled concentration of active drug, while the latanoprost active drug concentration degraded over the course of normal patient use in Sao Paolo, Brazil.

In the present study, the active drug concentration in all bimatoprost bottles tested was at least 100% of the labeled concentration more than 40 days after they were originally opened. Conversely, with latanoprost, only 8.8% of the bottles tested had at least 100% of the labeled active drug concentration. This result differs from a patient-use stability study conducted in Los Angeles, California, USA, where little degradation of latanoprost was found [10]. During the winter months when that study was carried out Los Angeles was in its wet season, in contrast to Sao Paolo's dry winter. Ambient temperatures were higher in Los Angeles during the study (21°C to 35°C), and patients may have been more likely to use air conditioning, and thus mitigate the effect of environment on the drug. Latanoprost has been shown to be sensitive to light and temperature [8,11], and variability in the actual conditions in which patients stored their medication may be a differentiating factor between the patient populations observed in these studies. A heat sensitive drug is more likely to degrade under improper storage, and these results might reflect variable storage practices from patient to patient and between study populations.

One limitation of the study was that baseline concentrations of the drugs were not measured prior to patient use. However, regulatory agencies and pharmaceutical manufacturing specifications require that actual drug concentrations be within a narrow range approximating the labelled strength. Approximately 10% more or less than the labelled concentration generally defines this tolerance, but it may be tighter depending on the characteristics of the drug [12]. The labelled concentration has been approved by regulatory agencies and produces the dose at which efficacy has been demonstrated. Actual concentration relative to the target label concentration, as presented here, gauges drug strength against approved, expected levels.

Whether the degree of drug degradation that occurs under normal-use conditions is clinically relevant is unclear. Clinical trials over 1 to 6 months have shown that bimatoprost produces IOP reduction greater than or comparable to latanoprost in various patient populations [3,13-17], and 2 meta-analyses support these conclusions [18,19]. Whether variable efficacy due to degradation over time could contribute to these differences has not been investigated, but future studies that correlate changes in concentration or degradation with efficacy measures may be of value.

Loss of drug activity may reduce IOP-lowering efficacy and cause wide fluctuations in a patient's IOP, which may be an independent risk factor for progressive disease [20,21]. Both bimatoprost and latanoprost reduce IOP over a range of doses [22-25]. Bimatoprost lowers IOP across a dose range from 0.001% to 0.1% in primates [24], and has been shown clinically effective at doses from 0.003% to 0.03% [25], with optimal IOP-lowering at the label dose of 0.03%. Likewise, latanoprost has been shown most efficacious at its label concentration of 0.005%, but still reduces IOP at 0.001% [22] and 0.0015% [23] concentrations. Even though lower drug concentrations may reduce IOP, drugs formulated at a lower concentration, as in the studies referenced above, may produce a different clinical response than a degraded medication. Selecting an IOP-lowering medication that is less sensitive to variation in environmental conditions may provide the best assurance of continued IOP control after the bottle is opened, thus reducing the risk of visual field loss.

Package inserts indicate that bimatoprost should be stored between 2°C and 25°C, and no shelf life limitation is indicated [26]. Latanoprost should be protected from light and refrigerated until opening, after which it may be stored at room temperature for 6 weeks [7]. Based on the number of drops dispensed from a 2.5 mL bottle, a bottle of bimatoprost may be expected to be in use for a maximum of 56 days, and latanoprost may last up to 47 days [27], which exceeds the 6 week recommendation for that drug. Patients who miss doses or otherwise extend the time a bottle of latanoprost is in use may exceed its recommended in-use life, increasing the risk of degradation and possibly reduced efficacy. Physicians should encourage their patients to observe expiration dates and storage instructions for all medications.

## Conclusion

Patients on ocular hypotensive drugs that consistently lower IOP are less likely to progress than patients whose medication levels decrease over time. In this study, latanoprost formulations degraded while bimatoprost did not, suggesting that patients are more likely to receive the prescribed dose of bimatoprost after opening. Patients that receive the labelled dose, which has been clinically shown to achieve maximal IOP lowering from that medication, are more likely to halt glaucomatous progression.

## Competing interests

MDP, NK and CCU declare that they have no competing interests. JGW is an employee of and owns stock in Allergan, Inc.

## Authors' contributions

MDP, NK, CCU collected the study medication bottles. MDP and JGW conceived of the study. All authors participated in the study design and coordination, and read and approved the final manuscript.

## Pre-publication history

The pre-publication history for this paper can be accessed here:


